# Neutron and X-ray diffraction study of ferrite nanocrystals obtained by microwave-assisted growth. A structural comparison with the thermal synthetic route. Corrigendum

**DOI:** 10.1107/S1600576714017452

**Published:** 2014-08-01

**Authors:** Eduardo Solano, Carlos Frontera, Inés Puente Orench, Teresa Puig, Xavier Obradors, Susagna Ricart, Josep Ros

**Affiliations:** a Departament de Química, Facultat de Ciències, Universitat Autònoma de Barcelona, Cerdanyola del Vallès, Barcelona 08193, Spain; b Institut de Ciència de Materials de Barcelona, ICMAB-CSIC, Campus de la UAB, Cerdanyola del Vallès, Barcelona 08193, Spain; c Instituto de Ciencia de Materiales de Aragón, ICMA-CSIC, Pedro Cerbuna 12, 50009 Zaragoza, Spain; d Institut Laue–Langevin, BP 156, 38042 Grenoble Cedex 9, France

**Keywords:** powder diffraction, ferrite, microwaves, nanoparticles, neutrons, structure, synthesis

## Abstract

In the paper by Solano *et al.* ▶ [*J. Appl. Cryst.* (2014), **47**, 414–420], Inés Puente Orench is missing from the list of authors. The complete list of authors should be Eduardo Solano, Carlos Frontera, Inés Puente Orench, Teresa Puig, Xavier Obradors, Susagna Ricart and Josep Ros.

## Abstract 2

Corrigendum to *J. Appl. Cryst.* (2014), **47**, 414–420.
